# Gun Shot Lesion of the Spinal Column1Read at the forty-second annual meeting of the Medical Association of Central New York, held at Auburn, October 19, 1909.

**Published:** 1910-04

**Authors:** J. P. Creveling

**Affiliations:** Auburn, N. Y.


### Gun Shot Lesion of the Spinal Column[1]

By J. P. CREVELING, M. D., Auburn, N. Y.

On September 26, 1908, there was admitted to the Auburn City Hospital, a patient with the following history:

Male, age 45, white, creamery worker by occupation; married but no children. Father committed suicide at the age of 65; mother died of phlebitis; one sister of phthisis pulmonalis and one brother of lead poison; one brother and five sisters living and well. At the age of 15 he contracted syphilis, for which he received no treatment for some six months, when a maculopustular skin eruption appeared which covered much of the body. He then began treatment and continued for the following three years. Since that time there has been no apparent return of the disease.

At the age of 37, he drank some ammonia water which produced stricture of the esophagus and connecting end of the stomach. He was operated upon and the trouble removed. Ten months before admission he contracted gonorrhea. He has anterior curvature of the dorsal spine and, has led a very intemperate life. When admitted to the hospital on the above date, he was in a semiconscious condition, bleeding from a self-inflicted bullet wound located a little to the left and slightly above the left nipple. He was very restless and complained of severe and constant pain in the left leg, which was entirely motionless. The pain seemed the principal factor that aroused him from his letharic condition. He remained in this state for three days, during which time there was retention of urine and feces. On the third day, a general left pleuritis developed which cleared up in something less than three weeks. The paralysis of the leg and pain, however, continued, the latter being sufficiently severe to require large and repeated doses of anodynes to afford any rest.

1. Read at the forty-second annual meeting of the Medical Association of Central New York, held at Auburn, October 19, 1909.

His general condition was becoming bad, the leg commenced to atrophy and the tendon reflexes were much diminished but not entirely lost. On October 23, he was radiographed to locate the bullet and, after a number of exposures, it was fixed as resting against the left laminæ of the last dorsal vertebra, or possibly involving the twelfth dorsal and first lumbar laminæ. Assuming that it might have fractured and compressed one of the laminæ, he was taken to the operating room and an incision to the left of the spinous processes down to the column was made, but both laminæ were found to be intact, and the bullet was not within the reach of the finger even after the wound had been extended so as to command the two last dorsal and first two lumbar vertebræ.

The wound was closed and he returned to the ward and was allowed to rest until December 16, when he was again skiagraphed. At this time a number of exposures were made by the senior house surgeon, and the location fixed as being imbedded in the body of the first lumbar vertebræ. Two days later he was again transferred for another effort at extraction. An incision some five inches long and parallel with the first was carried down to the transverse processes, which were removed by chisel as was also the twelfth rib. The bodies of the underlying vertebræ were exposed and the left half of the first lumbar cut away in anticipation of reaching the bullet imbedded in the bone. Failing in this, the corresponding portion of the second was similarly removed, and then the twelfth dorsal was treated in like manner.

This opened the spinal canal sufficiently to allow exploration and the bullet was found resting loosely between the body of the first lumbar and the spinal cord, suspended by loose inflammatory products. It was lifted out of its bed by a pair of forceps and removed. It had passed completely through the body of the twelfth dorsal and gravitated a fraction so as to rest opposite the upper margin of the first lumbar body. The lesion caused by the missile passing through the bony column was entirely repaired except, a small fragment that had been split off from the posterior surface of the vertebral body protruded into the canal. This of course was pressed into normal position. The covering of the cord seemed much engorged and rather changed in color. As stated, the cutting through the body of the vertebræ instead of the laminæ, was due to the supposition that, the projectile rested within that structure and not within the spinal cavity.

The wound was closed, which healed by primary union. He recovered from the anesthetic free from pain, and has remained so except, at intervals when it has returned in a much milder form. On the second day after the operation he could flex the leg, on the fourth day the leg upon the abdomen, on the eighth day he could stand upon his feet by the aid of crutches, and on the tenth could walk with the same aid. In two weeks time he had good motion in the leg, could stand without support and take a few steps unaided, by rigidly fixing the muscles of the back. Improvement has been steady until the present date.

The case is another illustration of the diagnostic value of the *x*-rays and surgically has some interesting features which will not be dwelt upon. The case illustrates the diagnostic value of the Röntgen ray and, at the same time the skiagraph demonstrates the liability to deception of exact location. In one picture the bullet seemed to rest against the vertebral laminæ, while in the other it appeared as if imbedded in the body of the vertebra.

Surgically, the case is of interest as it shows the extent to which the spinal canal can be opened and the bony structure removed, without disturbing the function of the cord; the rapidity with which symptoms of severe spinal lesion may disappear and how quickly the cord will resume normal action. I have not looked the subject up very exhaustively, but as far as I have done so I have been unable to find a similar case.

One that approaches nearest was treated by me some years ago, in which instance a large part of the second, third and fourth dorsal segments were removed for tubercular disease. This case was a boy 17 years old. It was not a primary operation as he had had a number of abscesses opened in the region of operation and I had excised the posterior half of one of the ribs sometime before. There was not much of an outlook for a favorable result, but at his solicitation the operation was done. The spinal cavity was not opened. Much of the body with more or less of the processes were removed. It was a long, bloody, tedious process, but his endurance was equal to the trial and he recovered sufficiently to leave the hospital without paralysis and in an apparently healthy condition of that portion of the back. He died however in less than a year of pulmonary tuberculosis.

In the first case the man is now at work in one of the shops in the city and I occasionally see him walking with his dinner pail in hand, to all appearances as well as before the injury.

22 South Street.

